# EXPOSURE OF PEDIATRIC EMERGENCY PATIENTS TO IMAGING EXAMS, NOWADAYS AND IN TIMES OF COVID-19: AN INTEGRATIVE REVIEW

**DOI:** 10.1590/1984-0462/2022/40/2020302

**Published:** 2020-12-18

**Authors:** Isabela Dombeck Floriani, Ariela Victoria Borgmann, Marina Rachid Barreto, Elaine Rossi Ribeiro

**Affiliations:** a Faculdades Pequeno Príncipe, Curitiba, PR, Brazil.

**Keywords:** Pediatrics, Emergencies, Diagnostic imaging, Medical overuse, Coronavirus infections, COVID-19, Pediatria, Emergências, Diagnóstico por imagem, Sobremedicalização, Infecções por coronavírus, COVID-19

## Abstract

**Objective:**

To analyze literature data about unnecessary exposure of pediatric emergency patients to ionizing agents from imaging examinations, nowadays and during times of COVID-19.

**Data sources:**

Between April and July 2020, articles were selected using the databases: Virtual Health Library, PubMed and Scientific Electronic Library Online. The following descriptors were used: [(pediatrics) AND (emergencies) AND (diagnostic imaging) AND (medical overuse)] and [(Coronavirus infections) OR (COVID-19) AND (pediatrics) AND (emergencies) AND (diagnostic imaging)]. Inclusion criteria were articles available in full, in Portuguese or English, published from 2016 to 2020 or from 2019 to 2020, and articles that covered the theme. Articles without adherence to the theme and duplicate texts in the databases were excluded.

**Data synthesis:**

61 publications were identified, of which 17 were comprised in this review. Some imaging tests used in pediatric emergency departments increase the possibility of developing future malignancies in patients, since they emit ionizing radiation. There are clinical decision instruments that allow reducing unnecessary exam requests, avoiding over-medicalization, and hospital expenses. Moreover, with the COVID-19 pandemic, there was a growing concern about the overuse of imaging exams in the pediatric population, which highlights the problems pointed out by this review.

**Conclusions:**

It is necessary to improve hospital staff training, use clinical decision instruments and develop guidelines to reduce the number of exams required, allowing hospital cost savings; and reducing children’s exposure to ionizing agents.

## RESUMO

**Objetivo:**

Analisar dados da literatura sobre exposição desnecessária de pacientes da emergência pediátrica a agentes ionizantes dos exames de imagem, na atualidade e em tempos de COVID-19.

**Fontes de dados:**

Entre abril e julho de 2020 ocorreu a seleção dos artigos, utilizando-se as bases de dados: Biblioteca Virtual em Saúde, PubMed e Scientific Electronic Library Online. Utilizaram-se os descritores: [(pediatrics) AND (emergencies) AND (diagnostic imaging) AND (medical overuse)] e [(Coronavirus infections) OR (COVID-19) AND (pediatrics) AND (emergencies) AND (diagnostic imaging)]. Incluíram-se artigos disponíveis na íntegra, em português ou inglês, publicados no período de 2016 a 2020 ou de 2019 a 2020, e artigos que contemplassem o tema. Excluiu-se artigos sem aderência com a temática e textos duplicados.

**Síntese dos dados:**

Identificaram-se 61 publicações, sendo 17 utilizadas para a elaboração desta revisão. Alguns exames de imagem utilizados nos Setores de Urgência e Emergência (SUEs) pediátricos, por emitirem radiação ionizante, aumentam a possibilidade de desenvolver malignidades futuras nas crianças. Destarte, há instrumentos de decisão clínica que possibilitam diminuir requisições de exames desnecessários, evitando a sobremedicalização e os gastos hospitalares. Ademais, com a pandemia da COVID-19, cresceu a preocupação com o uso excessivo de exames de imagem na população pediátrica, o que reafirma a problematização deste estudo.

**Conclusões:**

Veem-se como necessárias a capacitação da equipe hospitalar, a utilização de instrumentos de decisão clínica e a confecção de protocolos que possam avaliar a singularidade da criança. Isso permitirá reduzir o número de exames requeridos, possibilitando economia de custos hospitalares e redução da exposição de crianças a agentes ionizantes.

## INTRODUCTION

Computed tomography (CT), magnetic resonance imaging (MRI), X-ray and ultrasound (US) are widely used imaging examinations in Urgency and Emergency Services (UEs) for pediatric diagnosis and follow-up. However, these resources should be used carefully, together with the clinical judgment from health professionals, so that there is no overuse.^1^

Nowadays, due to technological advances, there has been an increase in the use of examinations, especially CTs. This is the physicians’ preferred choice in the UEs, because of the fast digitalization of images, which reduces the time of sedation in children.^2^ Therefore, there was an increase in the number of studies about the potential risks of exposure to ionizing radiation caused by imaging examinations, due to the tendency to develop genetic changes and future malignancies.^2^

On March 11, 2020, the World Health Organization (WHO) declared a pandemic caused by COVID-19.^3^ By July 9 of the same year, more than 11.8 million cases and 544 thousand deaths had been reported around the world.^4^ Even though infected children manifest less severe symptoms in comparison to adults,^5^ they can be hospitalized and exposed to ionizing radiation exams. Therefore, there is a growing concern about the potential overuse of imaging examinations in this population.

This review aimed to analyze literature data about the unnecessary exposure of pediatric emergency patients to ionizing agents from imaging examinations, nowadays and in times of COVID-19.

## METHOD

This is an integrative literature review (ILR) whose purpose is to synthetize and analyze studies that are available, from several methodological approaches, about the theme in question.^6^ Therefore, the identification of a large sample allows the evaluation, the critical discussion of the results and the development of a conclusion based on scientific evidence.^6^

To elaborate the research question, we used the PICO strategy - population, intervention, comparison and outcomes (Table 1). This review aims at answering: “What does the literature show about the unnecessary exposure of pediatric emergency patients to ionizing agents from imaging examinations, nowadays and in times of COVID-19?”. Then, we continued with the following stages of ILR: determination of databases, application of descriptors, and inclusion and exclusion criteria (Identification); analysis of titles and content of the abstracts of the identified articles (Screening); evaluation and critical inspection of studies in full (Eligibility); and definition of the analyzed articles for the confection of the IRL (Inclusion).^6^

**Table 1:** PICO Strategy: population, intervention, comparison and outcomes (results).

Criteria	Definitions
Population	Pediatric Urgency and Emergency Patients
Intervention	Exposure to ionizing agents of imaging examinations
Comparison	No intervention
*Outcomes*	Reduction of hospital costs and iatrogenesis (unnecessary exposure of the patient to ionizing agents to imaging examinations), nowadays and in times of COVID-19.

We selected the articles between April and July, 2020, using the following databases: Virtual Health Library, PubMed and Scientific Electronic Library Online (SciELO). The search descriptors were used in two stages, and were selected from the Health Science Descriptors (DeCS), combined in pairs based on the Boolean logic: AND or OR. In the first search, we used: (pediatrics) AND (emergencies) AND (diagnostic imaging) AND (medical overuse). In the second search, the following were used: (Coronavirus infections) OR (COVID-19) AND (pediatrics) AND (emergencies) AND (diagnostic imaging).

The search in the databases respected the following inclusion criteria: articles available in full, in Portuguese or in English, published from 2016 to 2020 (1^st^ search) or from 2019 to 2020 (2^nd^ search), and articles that contemplated the exacerbated use of imaging exams in pediatric emergency rooms. The exclusion criteria were: articles without adherence to the theme and duplicated texts in the databases.

**Figure 1:**
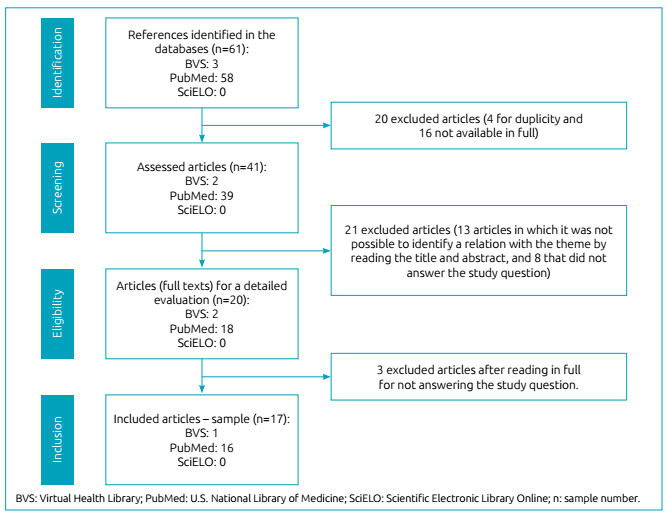
Flow of the selection process of articles for integrative review.

Sixty-one publications were identified, being three in the Virtual Health Library (5%), 58 in PubMed (95%), and none in SciELO (0%). In the identification stage, we excluded four texts due to duplicity, and 16 for not being available in full. Therefore, in the screening stage we analyzed 41 articles. Of these, after reading the title and abstract, 13 were excluded for not being related to the theme, and eight for not answering the research question. Thus, 20 articles were included in the eligibility stage; three were excluded after the texts were read in full, for not answering the research question. So, the final sample of this ILR comprised 17 articles (Figure 1).

For data extraction and analysis, we used a Microsoft Excel^®^ spreadsheet that included: authors, country of origin/year of publication, journal, study method and type of analyzed imaging examination.

The copyrights were respected by preserving the content exposed by the authors and by referencing the information extracted from the articles available in public domain.

## RESULTS AND DISCUSSION

Of the 17 analyzed articles, four were performed in the United States of America (USA); four, in China; two, in Italy; two, in Israel; two in the Republic of Korea; one, in Canada; one, in the Netherlands; and one, in Turkey. All articles were published in English. Regarding the year of publication, there was higher incidence in 2020 (seven articles), followed by 2018 (four articles), 2019 (two articles), 2017 (two articles) and 2016 (two articles). All of the analyzed articles contemplated the overuse of imaging examinations in pediatric emergency patients nowadays (Table 2) and in times of COVID-19 (Table 3). The analysis of the selected studies allowed the definition of seven categories.

**Table 2:** General characteristics of the included studies about the overuse of imaging examinations in pediatric emergency, nowadays.

Study	Study method (n)	Outcome
Gupta et al.^1^	Secondary instrument analysis (n=1,018)	The NEXUS instrument, for head CT, enabled the identification of pediatric patients with blunt trauma that really needed a CT, which can significantly reduce the use of this imaging examination in children.
Ohana et al.^2^	Review	Even though a reduction in the use of CT in children has been registered, the overuse rates are still very high. Therefore, it is necessary to implement protocols, such as PECARN and Alvarado, which regulate the requirements of imaging examinations in pediatric UE.
Rao et al.^7^	Retrospective study (n=207)	The use of the ACR Appropriateness Criteria guideline allowed pediatric patients submitted to neuroimage due to TBI to solve their symptoms with conservative treatment, thus reducing the number of unnecessary imaging examinations in children.
Reiter et al.^8^	Cohort study (n=505)	The intervention that reinforced the AAP guidelines in the pediatric emergency department led to the reduction of unnecessary imaging examinations, promoting higher cost-effectiveness and saving resources and time for the UE.
Chamberlain et al.^9^	Repeated cross-sectional analysis (n=3,313)	Pediatric UEs tend to present lower rates of X-ray requests compared to general UEs when treating children with acute exacerbation of asthma.
Kwon et al.^10^	Obsevational study (n=14,244)	There was a reduction in the total level of APF X-ray in children with gastrointestinal symptoms, after the adoption of a campaign with orientations about the examination.
Rawlins et al.^11^	Retrospective cohort study (n=592)	When requesting that the clinical staff of the UE consulted the Otorhinolaryngology team before requesting an imaging examination for children with unspecific physical examination, it was possible to observe reduction in the number of patients submitted to CT and the increase in the frequency of surgical interventions for the treatment of PTA.
Gökharman et al.^12^	Retrospective study (n=1,041)	With the PECARN instrument, it was possible to identify higher rates of appropriate CTs in pediatric patients with TBI, once this instrument has proven to enable the identification of the presence and severity of the pathology.
Broers et al.^13^	Retrospective multicenter cohort study (n=563)	The guideline from the Netherlands Society of Neurology (2010) is the orientation for the clinical management of pediatric patients with non-severe TBI in the UEs of hospitals in the Netherlands. However, there was a disagreement between this recommendation and clinical practice when observing the preference for hospitalization than the CT request.
Cohen et al.^14^	Retrospective study (n=23,591,084)	The rates of use of imaging examinations in the pediatric UEs were lower in Canada than in the USA. This lower rate is not associated to worse prognosis, suggesting that the USA can better administrate the use of resources and reduce, with safety, the performance of imaging examinations.

n: sample number; NEXUS: National Emergency X-Radiography Utilization Study; CT: computed tomography; PECARN: Pediatric Emergency Care Applied Research Network; UEs: Urgency and Emergency Service; TBI: Traumatic Brain Injury; AAP: American Academy of Pediatrics; APF: *abdominal plain film*; PTA: peritonsillar abscess; USA: The United States of America.

**Table 3:** General characteristics of the included studies about the overuse of imaging examinations in pediatric emergency, in times of COVID-19.

Study	Study Method (n)	Outcome
Musolino et al.^5^	Observational study (n=10)	The use of US to evaluate children with suspicion or confirmation of COVID-19 should be encouraged, considering that it allows the monitoring of pneumonia, reduces radiation and unnecessary sedation in pediatric patients, and reduces the risk of exposure to health professionals to SARS-CoV-2.
Zheng et al.^15^	Retrospective study (n=25)	While children present more favorable clinics and prognosis in COVID-19, in ages inferior to three years, represent critical cases of disease, which ratifies the need for special attention in care towards this population.
Shen et al.^16^	Single center and retrospective Study (n=9)	The clinical symptoms of the infection with SARS-CoV-2 in the pediatric population were atypical and had a less aggressive clinical course than in adults. To ensure the early diagnosis in children, in case of definitive diagnosis of infection in family members, the recommendation is that it is notified.
Xu et al.^17^	Review	Imaging examinations are important to identify and clinically manage COVID-19. The use of CT is recommended for the early evaluation of the infection, to the detriment of X-ray.
Huang et al.^18^	Ssytematic review and meta-analysis	Thoracic CT presents high sensitivity and low specificity in the detection of COVID-19, and should be performed in individuals with certain clinical characteristics, together with RT-PCR.
Cho et al.^19^	Retrospective review (n=6)	LUS has proven to be a fast and sensitive method as a screening tool to detect pneumonia and assess the severity of respiratory failure in hospitalized patients with COVID-19.
Smargiassi et al.^20^	Review	In pediatric patients admitted in the emergency room with symptoms similar to COVID-19, LUS should be performed early.

n: sample number; US: ultrasound; CT: computed tomography; RT-PCR: *reverse transcription-polymerase chain reaction*; LUS: lung ultrasound.

### 1st category - the relation between computed tomography and magnetic resonance imaging and the use of X-ray in pediatric emergency

In the retrospective study carried out in the USA,^7^ it was observed that the main imaging examinations used in the clinical investigation of pediatric patients are CT and MRI, both with its pros and cons, thus defining their utility. In comparison to MRI, CT tends to be cheaper, faster and more sensitive to bone fractures,^7^ besides presenting high diagnostic accuracy.^2^ However, a major disadvantage is the exposure of patients to ionizing radiation.^1^ ^,^ ^7^ On the other hand, MRI demonstrates to be less accessible, have higher costs and be less tolerable among younger children when compared to CT.^2^ ^,^ ^7^ However, It is a more favorable alternative regarding the reduction of radiation.^7^ That justifies the high number of studies performed in the past few years about MRI, in order to reduce the exposure of pediatric patients to ionizing agents.^2^

A cohort study performed in Israel^8^ points out that the thoracic X-ray is used for different respiratory emergencies, especially bronchiolitis, even if there is no recommendation for its use, according to the American Academy of Pediatrics (AAP).^8^ A similar orientation was identified in the North-American analysis,^9^ which does not advise the use of thoracic X-ray to treat acute exacerbation of asthma.^9^ The analysis conducted in the Republic of Korea^10^ stated there are many physicians using the X-ray in the screening of non-specific abdominal symptoms due to difficulties in diagnosis. Therefore, there is a flaw in relation to the recommendations defined in protocols and the reality of medical practice, which corroborates the exaggerated use of imaging tests in the pediatric population.

### 2nd category - the relation between ionization and possible malignancies associated with imaging examinations

An analysis conducted in the USA^1^ showed that CT is related to future risks of developing malignanies,^1^ ^,^ ^11^ especially in younger patients,^1^ for being an examination that uses ionizing radiation.

 Another study^9^ showed that, in childhood, the cells grow fast and become more prone to developing cancer when exposed to ionizing radiation. Besides, the small body area contributes with higher dose of accumulated radiation.^10^ It was estimated that 1.5-2% of malignant neoplasms in the USA may have been caused by radiation from CTs.^12^ Besides, it is assumed that CTs in the pediatric population may be related to 5,000 future annual cases of cancer.^11^

A study carried out in the Netherlands^13^ showed that the performance of a head CT increases the risk of developing a future brain tumor. This risk increases with the performance of additional CTs, and becomes even higher when the exposure involves children aged less than 5 years.^13^ For patients in this same age group, the assumption is that one case of leukemia will appear for every 5,250 head CTs.^1^

### 3rd category - the financial impact of the excessive use of imaging examinations in the hospital environment

In an administrative database analysis,^14^ there was a retrospective study including visits to pediatric UEs from 2006 to 2016, in four hospitals in Canada (1,783,753 visits) and 26 hospitals in the USA (21,807,332 visits). The observation was that the North-American and Canadian populations have different financial and care structural organizations.^14^ In both countries, professionals are paid for the number of provided medical services; however, Canadian doctors have a global view of hospital management and governmental budget restrictions. Therefore, there is a reduction in the total number of requested and provided services per year.^14^ Besides, in the USA there is a higher tendency of performing imaging examinations, even when there is no indication for it, in order to avoid the lack of documents in a possible lawsuit; unlike in Canada, which has 25% of lawsuits due to medical negligence, in comparison to the cases in the USA.^14^

Thus, the conclusion is that North-American physicians, in comparison to Canadian physicians, perform excessive procedures, which results in major increase in hospital expenses.^14^ A similar fact was identified in a retrospective study carried out in Turkey,^12^ which stated that the improper use of imaging examinations causes major economic losses for hospital UEs. Imaging examinations, when performed without necessity, generate an approximate cost of US$ 20 billion; the non-performance of improper imaging examinations, regardless of the type, could generate an annual saving of US$ 81 billion.^12^

### 4th category - the difference between hospitals in the use of imaging examinations in the pediatric Urgency and Emergency services

There is a tendency for UEs in general hospitals to request more imaging examinations than in exclusively pediatric hospitals,^9^ especially when it comes to CTs.^2^ Another variation occurs between teaching and non-teaching hospitals, as observed in a study carried out in the Netherlands.^13^ This difference was analyzed by the chi-square test, identifying fewer head CTs in two regional, non-teaching general hospitals (23.3 and 25.9%) in comparison to the teaching hospital (44.1%).^13^

A similar fact was observed in a Canadian study^14^ that compared the purposes of imaging examinations between pediatric emergency units in Canada and in the USA. The use of thoracic X-ray to handle bronchiolitis (absolute difference of 6.8%) and asthma (absolute difference of 0.7%) was lower in Canada, as well as the use of abdominal X-ray for constipation (absolute difference of 23.7%) and abdominal pain (absolute difference of 20.6%).^14^

### 5th category - instruments used to reduce the exposure to ionizing agents in pediatric emergency patients

Several instruments are used to guide health professionals as to the use of imaging examinations, in order to reduce the unnecessary exposure to ionizing agents in pediatric UE patients.^1^ ^,^ ^2^ ^,^ ^7^ ^,^ ^8^ ^,^ ^9^ ^,^ ^10^ ^,^ ^11^ ^,^ ^12^ The Alvarado Score is used for abdominal CT.^2^ For head CT, the following instruments were reported: Pediatric Emergency Care Applied Research Network (PECARN),^1^ ^,^ ^2^ ^,^ ^7^ ^,^ ^12^ National Emergency X-Radiography Utilization Study (NEXUS),^1^ ^,^ ^7^ Children’s Head Injury Algorithm for the Prediction of Important Clinical Events (CHALICE)^1^ and Canadian Assessment of Tomography for Childhood Head Injury (CATCH).^1^ ^,^ ^7^ The last two, however, were considered little reliable for clinical use due to their low sensitivity.^1^

The Alvarado Score^2^ is an instrument used for acute appendicitis and abdominal pain, in order to reduce and limit the use of CT, thus favoring the use of US and MRI. However, the adherence to this instrument was not effective in reference hospitals, unlike pediatric hospitals.^2^

The PECARN^1^ ^,^ ^2^ ^,^ ^7^ ^,^ ^12^ is a clinical decision instrument widely used in hospitals for the orientation of head CT in cases of traumatic brain injury (TBI).^2^ The focus of this tool is to identify patients with low risk of developing major clinical complications, without the need to being submitted to a CT.^2^ The PECARN not only has high sensitivity and low specificity,^1^ but it also has negative predictive value of approximately 100% for TBIs with high clinical relevance.^2^ Besides these characteristics, the PECARN can also be used to detect and classify the severity of pathologies, with 74.8% of sensitivity and 91.7% of specificity.^12^ The instrument allows to estimate the duration of hospitalization and, therefore, leads to the reduction of hospital resources and contact of the patient with radiation.^12^ This instrument presents excellent screening and is similar to medical judgment, so it is recommended by the AAP.^1^

NEXUS is a clinical decision instrument used for pediatric patients with TBI^1^ ^,^ ^7^ which aims at assisting physicians in the identification of low-risk patients, who do not need a CT request, and high-risk patients, who will require intervention.^1^ The great differential of this tool is the use of clinical judgment to conduct this risk stratification.^1^ This instrument properly classified all high-risk patients who should be submitted to neurosurgery, presenting a 100% sensitivity level.^1^ However, a study^1^ pointed out that the real sensitivity of the instrument is of 87.2%. Through the use of NEXUS, together with clinical judgment, the head CT requests decreased in up to 34% of low-risk pediatric patients.^1^

By comparing the PECARN and the NEXUS instruments, the conclusion was that sensitivity, in both cases, is similar, even though the analyzed samples are different.^1^ There is a difference in the way children can be classified regarding CT requests, considering that PECARN was developed for all patients with TBI, whereas NEXUS assesses only children previously determined by clinical judgment.^1^ This additional criterion of NEXUS directly implies on the reduction of unnecessary imaging examinations in approximately 10%.^1^

### 6th category - proposals of intervention

The need for using clinical judgment together with the guiding instruments of imaging examination requests was emphasized in order to optimize the diagnosis in pediatric UEs.^1^ These tools not only contribute with the knowledge of the medical professional, but also allow low-risk patients to be safely excluded from imaging examination requests, preventing overuse.^1^

The reimbursement policy for the quality of the provided service, and not for the number of procedures, can be an alternative to reduce imaging examinations and prescription of medication.^9^ It was suggested to audit and do collaborative benchmarking with the hospital staff in order to reduce examination overuse.^9^ Besides, UEs of general and pediatric hospitals can cooperate providing an integrated service for patients, by sharing pediatric guidelines.^9^ The training emergency programs of general hospitals should highlight the specificities of children in comparison to adults.^9^

Among the analyzed articles, some implemented effective proposals to reduce the use of imaging examinations. In the cohort study,^8^ an intervention that aims at limiting the use of thoracic X-ray to diagnose bronchiolitis and assess if pediatric UEs follow the current guidelines was implemented. Before intervention, the level of radiography was 44%, and then decreased to 36.6%. A similar reduction occurred in the hospitalization rates, which decreased from 76.8 to 69.8%. The conclusion was that the proposed approach was successful to reduce financial costs, pharmacological treatments and exposure to ionizing agents, as indicated in AAP guidelines. The authors suggested that similar interventions should be implemented in other pediatric UEs.^8^

A study^11^ analyzed the implementation of a policy whose purpose was to reduce the use of CT in patients with suspicion of peritonsillar abscess, recommending that professionals in the UEs request the evaluation of otolaryngologists before requiring imaging examinations in pediatric patients with unspecific physical examination. The efficacy of this policy was proven by observing a 13% reduction in the use of CT in the analyzed populations. This demonstrated that the evaluation by an expert, with clinical experience, reduces the number of unnecessary complementary examinations and leads to more accurate requests.^11^

### 7th category - use of imaging examinations in pediatric patients in times of COVID-19

The infection by SARS-CoV-2 in pediatric patients has shown milder, non-typical symptoms, with lower mortality rates, in comparison to adult patients.^5^ ^,^ ^15^ ^,^ ^16^ ^,^ ^17^ One of the analyzed explanations is the fact that the immune system of children is still immature, which leads to reduced inflammatory effect - and lower cytokine release - and, consequently, lower clinical expression.^16^

Imaging examinations are essential for diagnosis and for the early detection and monitoring of COVID-19.^5^ ^,^ ^17^ CT is a widely used instrument in the investigation of the infection,^5^ ^,^ ^17^ ^,^ ^18^ ^,^ ^19^ even though it does not distinguish it from other viral pneumonias.^17^ However, CT exposes patients to unnecessary radiation, and health professionals, to higher risk of cross contamination in the hospital.^18^ ^,^ ^19^ Thus, the American College of Radiology, in March 2020, advised against the use of this examination as a primary diagnostic method.^18^ However, CT should be chosen in some clinical situations, together with the reverse transcription-polymerase chain reaction (RT-PCR).^18^

Thoracic X-ray presents low sensitivity and specificity in the detection of pneumonia caused by COVID-19.^5^ On the other hand, CT is better for detecting changes in the early stage of the disease.^17^ For these reasons, it is the alternative of choice in relation to thoracic X-ray, suggested by some radiology societies.^5^ However, a retrospective study from a Chinese center,^16^ which analyzed nine pediatric patients with COVID-19, demonstrated that most CTs did not show changes; only two children had minor unilateral ground-glass opacities. In a Chinese retrospective study^15^ that analyzed 25 infected children, 24 were submitted to CT; of these, eight (33.3%) did not present radiological changes. These disparate data suggest that further studies are necessary to verify the reliability of the use of CT in the infected pediatric population.

Studies^5^ ^,^ ^19^ ^,^ ^20^ showed that lung ultrasound (LUS) is a reliable alternative for the diagnosis of the new coronavirus. One advantage of LUS is that it is more sensitive than thoracic X-ray^19^ and it does not expose children to the ionizing radiation present in other imaging examinations.^5^ ^,^ ^19^ LUS is possible to be performed by the medical team in the bed side, reducing the risks of cross contamination.^5^ ^,^ ^19^ It also provides reliable data for evaluation, diagnosis and clinical follow-up of acute respiratory failure.^19^ When the pediatric patient is admitted to the UEs, with symptoms suggestive of the new coronavirus and visible lung impairment at LUS, there is a high chance that the child has viral pneumonia.^20^ So, the examination can be used as a standardized tool to perform differential diagnoses^20^ and the early evaluation of patients with suspicion of COVID-19.^5^

Therefore, it is necessary to establish guidelines for pediatric cases of infection by the new coronavirus, in order to prevent the overuse of examinations in this population.^19^ Besides, it is important to train doctors from different specialties to recognize the pathological findings of LUS and to store the results in a database, in order to create, in the future, an automatic algorithm to identify these echographic patterns.^20^ However, the use of other imaging examinations should not be ruled out,^5^ ^,^ ^19^ such as CT and thoracic X-ray, considering the fast evolution of the SARS-CoV-2 infection^17^ and the different clinical staging of the disease.^5^

This review allowed to identify that, nowadays, there is a tendency for the exacerbated use of imaging examinations in pediatric patients in UEs. Therefore, it is necessary to train the hospital clinical staff, the use of clinical decision instruments and the confection of efficient protocols that can assess the singularity of the child. This will allow short and long-term benefits: reduction in the number of examination requests, enabling to save in hospital costs, reduce the exposure of pediatric patients to ionizing agents, considering that these can cause future malignancies.

Because of the infection by the new coronavirus, strategies are necessary so that there is no overmedicalization in the pediatric population. One of them is the creation of guidelines that limit the use of examinations with ionizing radiation and favor the use of LUS. Therefore, it is possible to gather, afterwards, a database of characteristic ultrasound and radiological findings to facilitate the diagnosis of infection by SARS-CoV-2.

The analyzed studies allowed to recognize the importance of this theme and its global diffusion, especially in North-American, European and Asian continents. However, there were no Brazilian studies about the theme, and its conduction is recommended to follow up the tendencies of international research and validation of the aforementioned instruments.
